# Reconsidering the evolution of brain, cognition, and behavior in birds and mammals

**DOI:** 10.3389/fpsyg.2013.00396

**Published:** 2013-07-01

**Authors:** Romain Willemet

**Affiliations:** UnaffiliatedToulouse, France

**Keywords:** allometry, behavior, brain evolution, cerebrotypes, cognition, general intelligence, mentality, personality

## Abstract

Despite decades of research, some of the most basic issues concerning the extraordinarily complex brains and behavior of birds and mammals, such as the factors responsible for the diversity of brain size and composition, are still unclear. This is partly due to a number of conceptual and methodological issues. Determining species and group differences in brain composition requires accounting for the presence of taxon-cerebrotypes and the use of precise statistical methods. The role of allometry in determining brain variables should be revised. In particular, bird and mammalian brains appear to have evolved in response to a variety of selective pressures influencing both brain size and composition. “Brain” and “cognition” are indeed meta-variables, made up of the variables that are ecologically relevant and evolutionarily selected. External indicators of species differences in cognition and behavior are limited by the complexity of these differences. Indeed, behavioral differences between species and individuals are caused by cognitive and affective components. Although intra-species variability forms the basis of species evolution, some of the mechanisms underlying individual differences in brain and behavior appear to differ from those between species. While many issues have persisted over the years because of a lack of appropriate data or methods to test them; several fallacies, particularly those related to the human brain, reflect scientists' preconceptions. The theoretical framework on the evolution of brain, cognition, and behavior in birds and mammals should be reconsidered with these biases in mind.

## Introduction

Despite decades of research, some of the most basic issues related to the evolution of brain, cognition, and behavior are still unclear. In particular, in birds and mammals, in which most research has been conducted, there are no satisfactory answers to the following questions: Which factors control species differences in brain size and composition and what is, if any, the role of body size? How do brain size and composition influence species behavior? What is the nature of species differences in cognition and behavior? As a matter of fact, the increasing number of hypotheses being proposed on evolutionary neuroscience and comparative cognition is at odds with the paucity of theoretical background. For example, there is a widespread belief that “although absolute brain size may partially explain species differences in intelligence, the fact that elephant and whale brains are several times larger than human brains gives us pause and suggests the need to control for body size” (Rilling, 2006). Yet, is it possible to compare species' brains irrespective of their taxa? Is it justified to consider body size as the main factor controlling brain size? Finally, should the human brain be taken as the reference brain when considering cognitive abilities? As discussed in this paper, the answers to these questions are negative.

More generally, there is a need for reconsidering some of the methodological and conceptual bases of comparative neuroscience. Examining the literature on birds and mammals, the present paper exposes definitive reasons for abandoning whole-class analyses in comparative studies and highlights the importance of a detailed taxon-cerebrotype approach in brain evolution studies. Possible factors underlying the changes in brain size and composition inside a taxon-cerebrotype are presented, as well as potential factors associated with variation in relative brain size. These results are then summarized in a methodological section on measuring cognitive abilities between species and in a section about species variations in cognition and behavior. The final section discusses the significance of intra-species scaling in the evolution of brain and behavior.

## Comparative brain studies in birds and mammals

In a sample of species ranging from bacteria and viruses to whales and sequoia, there is a fairly strong relationship between species size and generation time or population density (Harvey and Pagel, 1991, 3–4). Yet, because reproduction mechanisms as well as the mechanisms underlying population density differ between the taxonomic groups included in the sample, comparative analyses on the factors underlying such relationships are inappropriate at this phylogenetic level. This difficulty has generally been overlooked in evolutionary neuroscience, leading to some misleading concepts to be accepted and continuously reported. The encephalization quotient (EQ) approach developed by Jerison (1973) is an example of this concern. Basically, because brain size appears to increase regularly with body size by a power law in mammals, Jerison has suggested the possibility of calculating the expected brain mass of any species from its body mass and relating it to the processing capacity by the mean of the EQ (a measure of observed brain size relative to the expected brain size predicted by the brain/body power law). As betrayed by its frequency in literature, the very principle of the EQ method has been widely accepted. Yet, two major flaws, independently fatal for the EQ approach, are presented below (see also Herculano-Houzel, 2011a).

### Brain/body scaling differences

**Figure 1:**
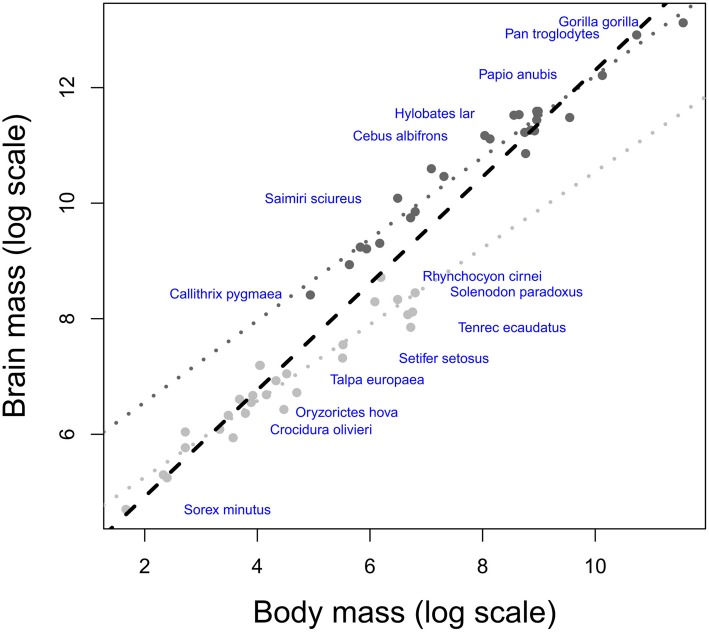
**Scatter plot of brain weight onto body weight (log scale) in insectivores (light gray) and simians (dark gray)**. Humans have been excluded from the analysis due to their large encephalization quotient (Rilling, 2006; but see Herculano-Houzel and Kaas, 2011). The dotted lines represent the respective brain/body allometric slopes for each taxon, and the black dashed line represents the common allometric slope. Note that when using a common slope; the largest brained simian species possess negative residuals, whereas the smallest brained insectivore species possess positive residuals.

The minimal assumption for the EQ method to be valid is a universal exponent describing the relationship between brain and body size among mammals. Yet, such an exponent is theoretically implausible and not supported by empirical results (Worthy and Hickie, 1986; Harvey and Krebs, 1990). Thus, there is no “expected brain size” for any mammalian species because brain/body allometry is specific to each taxon. Besides, this suggests that the extensive debate on the value and significance of a hypothetical mammalian exponent [see review by Harvey and Krebs (1990)] is groundless. Such taxa-specific brain/body allometries, also present in birds (Mlikovsky, 1989; Nealen and Ricklefs, 2001), are widely acknowledged, but their effects largely underestimated (see also Deacon, 1990a). Yet, this causes a systematic bias in comparative analyses, visible for example when regressing brain and body size from simian and insectivore species [Figure 1, data from Stephan et al. (1981)]. All statistical analyses in this paper have been performed using R software (R Development Core Team, 2011).

### Taxon-cerebrotypes

**Figure 2:**
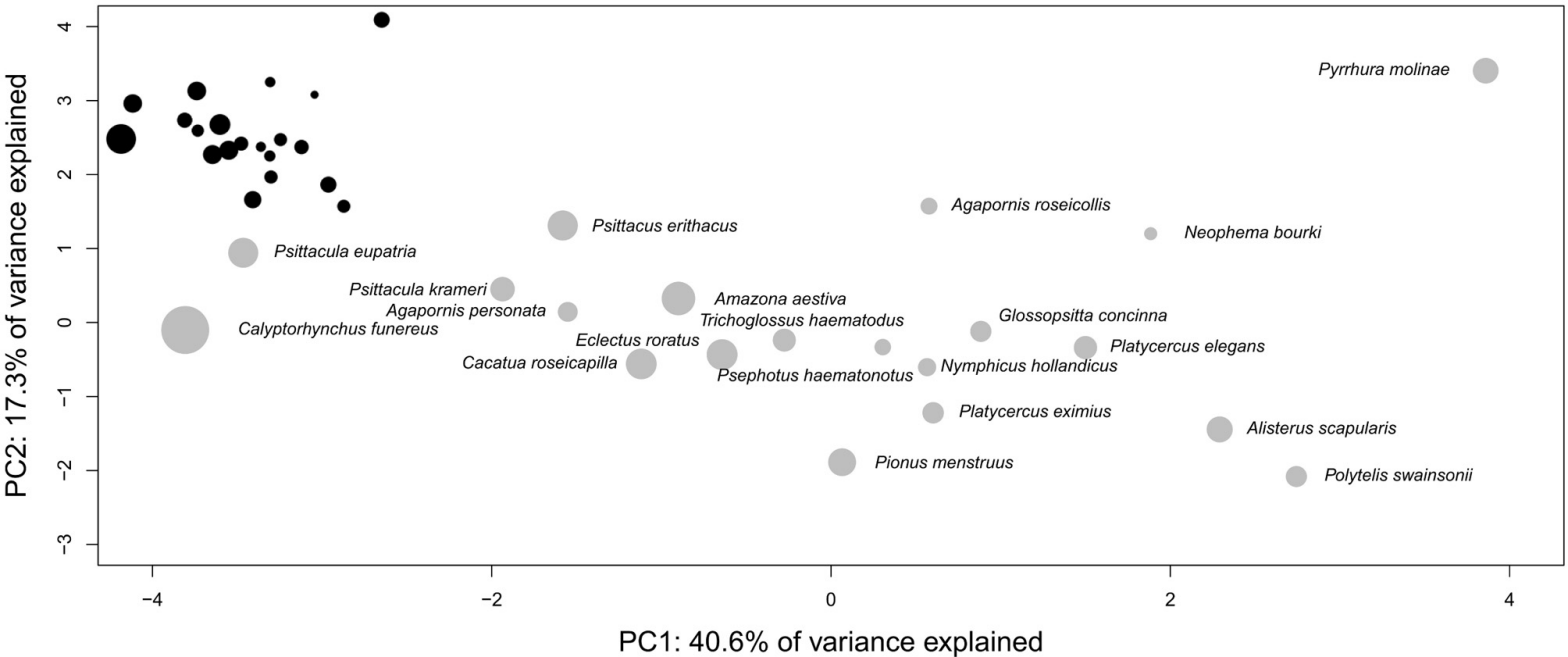
**Three dimensions principal component analysis of the proportional size of Psittaciformes brain structures [data from Iwaniuk and Hurd (2005), the analysis includes the size of nidopallium, Wulst, mesopallium, striatopallidal complex, remainder of the telencephalon, optic tectum, cerebellum, diencephalon, and brainstem]**. The diameter of the discs indicates the relative size of the brains in the taxon, i.e., the species with the biggest brain in the taxon possesses the largest disc, and the area of the disc of all the other species is proportional to the volume of their brains compared to the volume of the biggest brain in the taxon. The black figure at the top left represents the same pattern at scale 1:1 between x and y axes. Although absolute brain size (log) significantly correlates with position on PC1 (*t* = −2.754, *df* = 18, *p*-value = 0.01306, cor = −0.54), the relationship is driven by a single species (*Calyptorhynchus funereus*). Removing it from the analysis confirms the absence of correlation between brain size and the position in the PCA plan suggested by the figure (*t* = −1.7705, *df* = 17, *p*-value = 0.09456, cor = −0.39).

Studies using the EQ assume that brain size is an estimate of processing capacity (Jerison, 1985). Such a hypothesis would require that mammalian brains are size variations of the same model; an issue tackled by the debate on whether the evolution of mammalian brain structures has been limited by some developmental constraints (“concerted evolution,” Finlay and Darlington, 1995; Finlay et al., 2001), or whether the size of brain structures could vary independently between species (“mosaic evolution”, Barton and Harvey, 2000). In fact, it is possible to define groups of brains that have evolved under a bauplan that differs from those of other taxa at most anatomical levels (“taxon-cerebrotypes” Willemet, 2012 after Clark et al., 2001). Such diversity, despite the presence of developmental [review in Charvet and Striedter (2011)] and functional (Kaas, 2000) constraints definitely undermines the notion of a “universal scaling law” (Jacobs, 2012) in the evolution of the mammalian brain structures (Willemet, 2012). Consequently, “the fact that elephant and whale brains are several times larger than human brains” (see Introduction) is, by itself, uninformative on their respective cognitive abilities. Iwaniuk and Hurd (2005) have shown that bird species could also be grouped into taxon-cerebrotypes (following Clark et al., 2001). Interestingly, Figure 2 suggests that, unlike mammalian taxa, the brain composition of Psittaciformes species is not predicted by their brain size. The range of brain size (13-folds between the smallest and largest brain of the Psittaciformes dataset, compared to 18,100 and 250-folds in Pteropodidae, simians (human species excluded) and carnivores, respectively, data from Reep et al. (2007), Stephan et al. (1981) and unpublished data from the same research group) is maybe too small to make a tendency visible, or species variability in Psittaciformes is particularly large. Should this observation be confirmed and generalized to other bird-cerebrotypes, it would suggest that both the factors underlying brain evolution inside a taxon and the methods needed to study them might differ between birds and mammals.

### Remarks

The EQ method has been used here because of its significant influence on brain studies and its popularity outside the scientific community. The problems highlighted above should affect every analysis in which datasets exhibit taxa-specific relationships between variables (for instance in the scaling of the basal metabolic rate with body size, (White et al., 2009, or the duration of the gestation period, Martin et al., 2005). Studies on brain composition at the bird or mammalian level will necessary produce inconsistent results [see for example Kaskan et al. (2005) and Barton (2007) for opposite conclusions concerning the adaptation of the visual system in mammals and primates, respectively]. Indeed, even traditional groups such as bats, cetaceans, or primates are potentially subject to these inter-taxa characteristics (e.g., Willemet, 2012). Comparative methods such as phylogenetically independent contrasts (PIC, Felsenstein, 1985) are ineffective in dealing with this issue. The solution comes from the determination and analysis of taxon-cerebrotypes (Willemet, 2012).

## Comparing taxon and species cerebrotypes

### Taxon-cerebrotype differences

**Figure 3:**
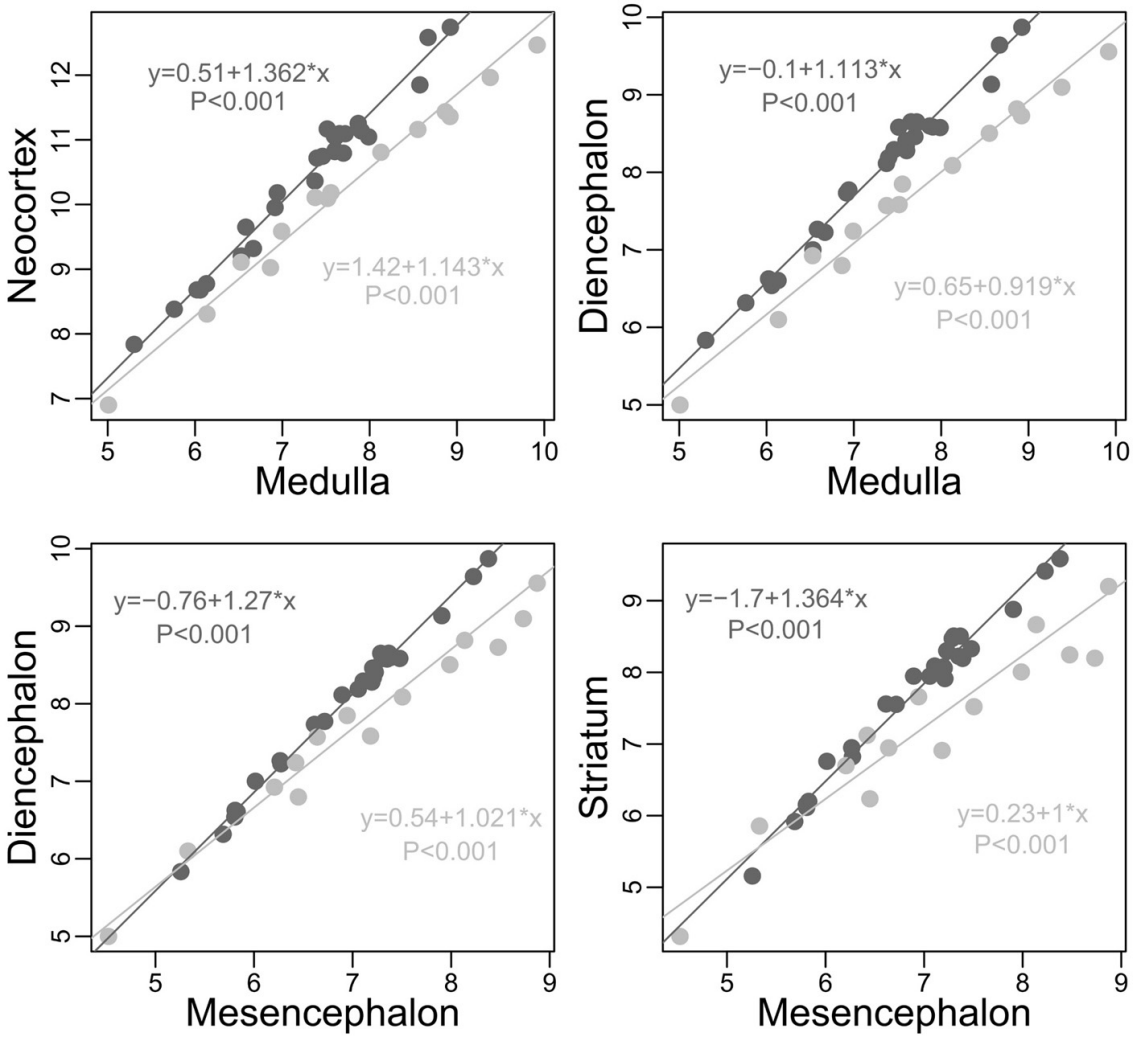
**Plots of some of the most obvious differences between carnivores (light gray) and simians (dark gray, human species excluded) cerebrotypes (log scale)**. On average (although an average is inappropriate due to slope differences), for similar medulla sizes, simians possess a neocortex and a diencephalon 2 times larger than carnivores (neocortex: Minimum 1.323, 1st Quartile 1.506, Median 2.007, Mean 2.047, 3rd Quartile 2.322, Maximum 3.509; diencephalon: Min. 1.362, 1st Qu. 1.611, Median 2.001, Mean 1.981, 3rd Qu. 2.150, Max. 2.785). On average again, for similar mesencephalon size, simians possess a diencephalon and a striatum, respectively, 55 and 85 percent larger than carnivores (diencephalon: Min. 0.9346, 1st Qu. 1.3890, Median 1.5660, Mean 1.5480, 3rd Qu. 1.7180, Max. 2.1880; striatum: Min. 0.7174, 1st Qu. 1.5750, Median 1.8810, Mean 1.8500, 3rd Qu. 2.2310, Max. 2.6490). Slope differences between these two taxa, unobserved in Willemet (2012), are clearly visible here.

**Figure 4:**
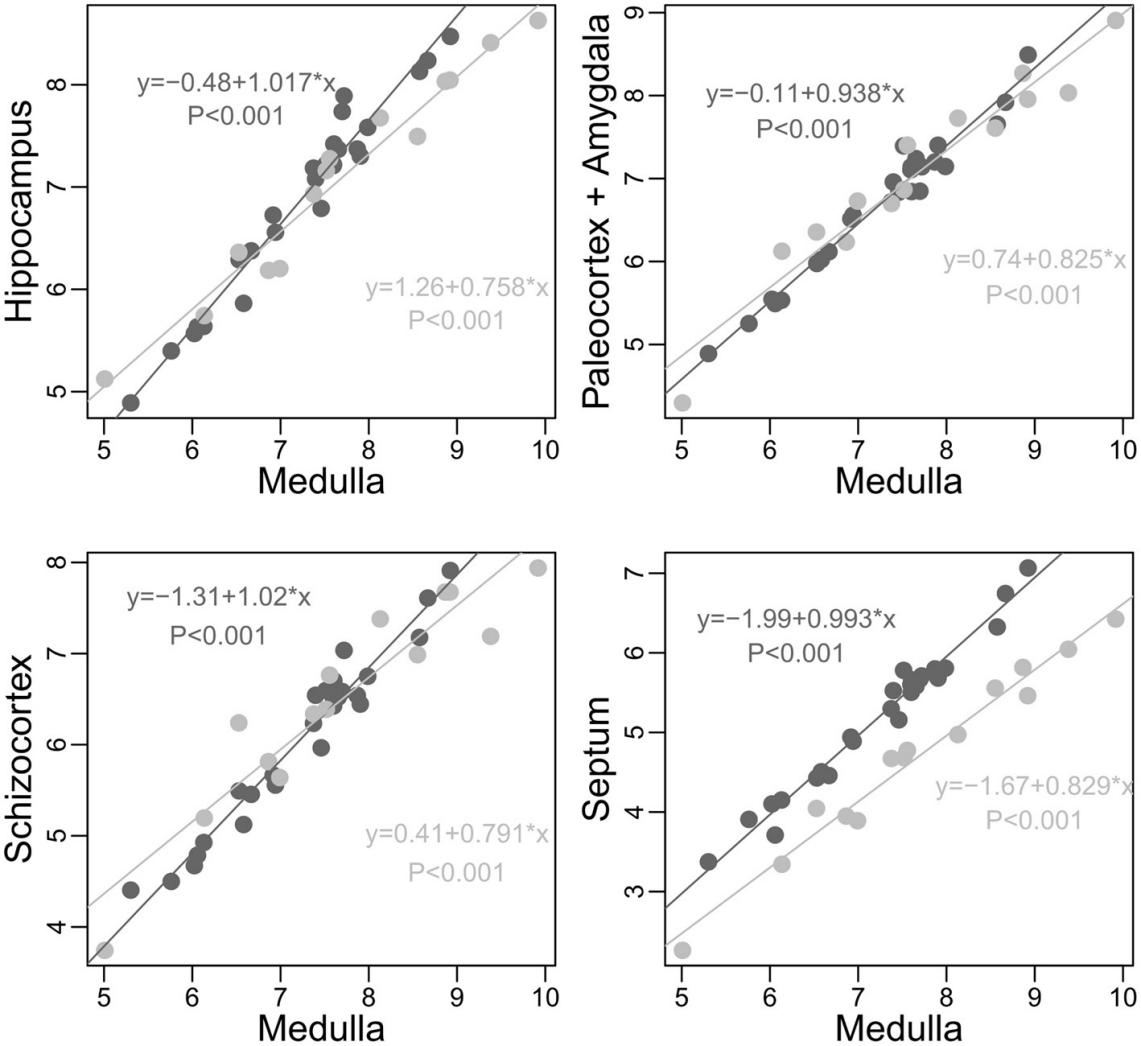
**Scaling of limbic structures onto medulla (log scale) in carnivores (light gray) and simians (dark gray)**. To strengthen the point, pinnipeds species are not included in this analysis (pinnipeds apparently have smaller hippocampus, paleocortex, and amygdala than fissipeds of similar medulla size, but similar septum and schizocortex size, unpublished analysis). In no cases does simians as a group have smaller limbic structures than predicted by the size of their medulla when compared to carnivores (differences between the actual size of the structure and the expected size predicted by carnivore allometry; hippocampus: Min. 0.6793, 1st Qu.0.9240, Median 1.2180, Mean 1.2010, 3rd Qu. 1.3930, Max. 2.1860; paleocortex: Min. 0.7696, 1st Qu.0.8467, Median 0.9241, Mean 0.9918, 3rd Qu.1.1050, Max. 1.5790; schizocortex: Min. 0.6056; 1 st Qu.0.7367; Median 0.9523; Mean 0.9787; 3rd Qu. 1.0800; Max. 1.6810; septum: Min. 1.434, 1st Qu. 2.119, Median 2.383, Mean 2.442, 3rd Qu. 2.646, Max. 3.830). See also Table 1.

For morphological and sensorial reasons, the quantity of information coming from and directed to the body should differ between taxa. Also, for ecological reasons, the nature of the information from the environment and the manner of analysing it should differ between taxa (see section Factors Underlying the Evolution of the Size and the Composition of Brains). Accordingly, an analysis comparing the size of each structure relative to the other structures in carnivores and simians (Table 1 and Figure 3, see also Figure 4) reveals significant differences between these two taxon-cerebrotypes [simian data from Stephan et al. (1981); carnivore data from Reep et al. (2007)]. Moreover, and in addition to the fact that simian species possess a brain around twice as large as carnivore species of similar body weight [data from Kirk (2006) and Finarelli and Flynn (2009)], a carnivore brain differs from a simian brain in many other characteristics. For example, except for small species, simians have a bigger frontal cortex than carnivores for a similar size of the rest of the neocortex (Bush and Allman, 2004, the term “neocortex” has been preferred here against “isocortex” following Butler and Hodos, 2005). Also, carnivore and simian cerebrotypes differ in the pattern of neocortex girification (Pillay and Manger, 2007) and cortical layering (Hutsler et al., 2005). In fact, it is likely that differences between these two taxon-cerebrotypes affect most neuroanatomical levels. Distinguishing taxon-cerebrotypes and species-cerebrotypes differences is fundamental in understanding how brains control species differences in cognition and behavior. In this regard, the nuclear arrangement of the avian pallium offers an interesting evolutionary alternative to the mammalian neocortex (Güntürkün, 2005, 2012). It is important, however, not to systematically generalize observations from one taxon-cerebrotype to others (Striedter, 2002).

**Table 1:** **Differences between carnivore and simian cerebrotypes (human species excluded)**.

	**Medulla**	**Cerebellum**	**Mesencephalon**	**Diencephalon**	**Striatum**	**Septum**	**Paleocortex**	**Hippocampus**	**Schizocortex**	**Neocortex**
Medulla		^***^	^***^	^***^	^***^	^***^	.	^**^	NS	^***^
Cerebellum	(+)		.	^***^	^***^	^***^	^*^	NS	.	^***^
Mesencephalon	(+)	−		^***^	^***^	^***^	NS	.	NS	^***^
Diencephalon	(+)	(+)	(+)		^*^	^***^	^***^	^*^	^***^	^***^
Striatum	(+)	(+)	(+)	(+)		^**^	^***^	^**^	^***^	^***^
Septum	(+)	(+)	(+)	(+)	(+)		^***^	^***^	^***^	^***^
Paleocortex	+	(−)	+	(−)	(−)	(−)		^**^	NS	^**^
Hippocampus	(+)	+	+	(−)	(−)	(−)	(+)		^***^	.
Schizocortex	+	−	−	(−)	(−)	(−)	−	(−)		^***^
Neocortex	(+)	(+)	(+)	(−)	(−)	(−)	(+)	+	(+)	

First, a regression of a structure Y (rows) onto another structure X (columns) is done for simian species (log scale). Second, carnivore residuals for the structure Y are calculated using the equation obtained previously for simians and the carnivore structure X. Third, differences between carnivore and simian residuals are tested using a Mann–Whitney test (if there is no difference between the two groups, mean carnivore residuals = mean simian residuals = 0). The symbols on the upper diagonal indicate the level of significance:

*** < 0.001;

** < 0.01;

* < 0.05;. < 0.1; NS > 0.1. On the lower diagonal, a plus sign indicates that simians generally have more Y than carnivores for similar X size, while a minus sign indicates the opposite. Brackets around the signs indicate significance and are redundant with the symbols on the upper diagonal. For further details, see Figures 3 and 4. Note: removing Pinniped species (see below) does not affect the general conclusions. In all cases, this method is illustrative only, as larger datasets and finer analyses are required before definitive conclusions. The simian species include: Alouatta seniculus, Aotus trivirgatus, Ateles geoffroyi, Callicebus moloch, Callimico goeldii, Callithrix jacchus, C. pygmaea, Cebus albifrons, Lophocebus albigena, Cercopithecus ascanius, C. mitis, Piliocolobus badius, Erythrocebus patas, Gorilla gorilla, Hylobates lar, Lagothrix lagotricha, Macaca mulatta, Miopithecus talapoin, Nasalis larvatus, Pan troglodytes, Papio anubis, Pithecia monachus, Pygathrix nemaeus, Saguinus midas, S. oedipus, Saimiri sciureus. The carnivore species include: Bassaricyon gabbii, Callorhinus ursinus, Canis latrans, Crocuta crocuta, Eumetopias jubatus, Mephitis mephitis, Mustela nivalis, Nasua nasua, Panthera leo, P. pardus, Phoca vitulina, Procyon cancrivorus, Puma concolor, Taxidea taxus, Ursus maritimus, Vulpes vulpes, V. zerda, Zalophus californianus.

### Methodological issues

There are several difficulties inherent in allometric analyses. First, researchers must distinguish between characters (potentially) shared by all mammalian species and taxa or species-specific evolutionary specializations. A particularly compelling example of this issue is the spindle like cells that were originally thought to be unique to apes and humans (Nimchinsky et al., 1999), but that have then been discovered in the large brained species of virtually all mammalian taxa (Butti et al., 2011). Second, researchers must distinguish between the confounding effects of the relative and absolute size of the structures. For example, Reep et al. (2007) hypothesized that “perhaps the reduction of the olfactory system in primates and humans is the unfortunate but tolerable result of selection to increase the size of the isocortex by reassigning stem neurons from olfactory bulb and hippocampus to the isocortex” (see also Yopak et al., 2010). But do primates (or simians) possess relatively small limbic structures? It actually depends on *relative to what* (Figure 4, see also Smith and Bhatnagar, 2004 and Heymann, 2006 for interesting discussions on olfaction in primates). In fact, the apparent trade-off described by Reep et al. (2007) is due to differences in the structure relative sizes. Although in simians, the enlargement of the neocortex (and cerebellum) has largely outweighed the enlargement of other structures, it does not constitute proof of a “push-pull relationship”, since there is no evidence that the selection of the limbic structures has been constrained by the selection of a large neocortex. Therefore, understanding the differences between species and taxon cerebrotype requires the use of precise methods, able to examine the variations of single brain structures and systems, at the species and group level.

## Factors underlying the evolution of the size and the composition of brains

Although understanding the factors underlying species differences in brain size and composition is a key issue in evolutionary neuroscience, our current understanding of it is limited. Nowadays, the “passive growth” of Aboitiz (1996) is considered to be “the main determinant of brain size across species” (Aboitiz, 2001). Under this view, the “brain increases in size by virtue of genetic and developmental coupling with the rest of the body”; a process that “does not necessarily imply higher processing capacity” (Aboitiz, 2001). Striedter (2006) suggested that “evolutionary changes in absolute brain size […] are an ‘automatic’ consequence of changing body size” (original quotation marks) and that “increasing absolute brain size ‘automatically’ changes the proportional size of the individual brain regions” (Striedter, 2006, original quotation marks). Under this view, the evolutionary significance of brain structure size in terms of cognitive abilities would be at best indirect, as its evolution would depend on factors others than those selecting for cognitive abilities. Indeed, Deacon (1990a) considered that “brain size […] is at best a hypothetical correlate of some poorly understood mental parameters (e.g., intelligence, memory), which themselves are only hypothetically correlated with any ecological parameter and are highly canalized and inflexible in development.” More recently, referring to the large proportion of the neocortex in big brained mammalian species, Barton suggested that “whilst it might be tempting to speculate on the hitherto unappreciated intelligence of these species, the most parsimonious explanation is that they are just large animals” (Barton, 2012). Yet, instead of being strongly anchored in an empirical and theoretical framework, such a view arose from misconceptions on the significance of allometric relationships. Firstly, the fact that two variables (here, brain and body size) allometrically correlate does not imply that one variable (body size) controls the other (brain size). Second, brain size is no more than the cumulative size of the structures that constitute it. Therefore, it is probably more correct to consider that brain structure sizes control brain size, rather than the reverse. In this section, and even though much more work is needed to understand the factors influencing size variations of each brain structure (thereby controlling both brain size and composition), several factors playing a role in the selection of brain structures (in particular the neocortex and cerebellum) are presented.

### Somatic factor

Even though the correlation between brain and body size in mammals and birds has long been recognized (e.g., Dubois, 1897), the reasons for this relationship have, as emphasized above, always remained unclear. An obvious but important factor is that the brain must fit into the skull (Striedter, 2005), so that only large animals can support large brains. However, this fact alone cannot explain why large animals often have large brains. It has been hypothesized that “larger organisms, which generally have larger organs, need to have larger brains in order to control and service the increasing somatic and vegetative demands that are inevitable consequences of increases in body size” (Hofman, 1989). In agreement with this hypothesis, spinal cord size rises with body size in mammals (MacLarnon, 1996). This “somatic factor” (Jerison, 1973) is the first reason for which we can expect a concerted pattern of evolution inside taxon-cerebrotypes. Consistent with this approach is the fact that brain size scales more tightly with fat-free body weight than fat weight (a poorly innervated tissue) in a dataset of 19 rodent species (Schoenemann, 2004). This hypothesis seems valid for the structures primarily implied with somatic function, but not sufficient for explaining the enlargement of the neocortex and cerebellum in particular.

Stevens (2001) has shown that, in primates, the number of neurons (and the size) of the primary visual cortex scales with a positive allometry compared to the number of neurons of the lateral geniculate nucleus because as eyes get bigger in species, the linear resolution of distances in the visual world also increases, and in order to maintain the same spatial resolution in the neocortex, the number of cortical neurons must increase with positive allometry compared to the neuron number in the lateral geniculate nucleus (the visual center of the thalamus). Thus, part of the coordinate increase between brain and body size could be related to this kind of functional constraints (see also Collins et al., 2013). A study of the allometric relationship at the cellular scale of the primate's brain and spinal cord indicates that although brain mass increases linearly with cord mass, the number of neurons in the brain increases faster than the number of neurons present in the spinal cord (exponent of 1.7, Burish et al., 2010). While this result could be consistent with the functional hypothesis presented above, some associative areas of the brain exhibit a positive allometry compared to other areas in primates (for example the prefrontal cortex, e.g., Smaers et al., 2011a). This suggests that other factors participate in the enlargement of structures.

### Non-somatic factors

Although it is clear that no researcher ever considered the brain just as the body's control center, most authors have adopted methods that explicitly consider the somatic factor as the most important function of the brain. For example, residual analyses or multiple regressions are used to control for “body size effects” (e.g., Iwaniuk et al., 2004), and most authors would agree that “the strongest driver of brain size is body size” (Changizi, 2010). However, brains guide individuals inside their ecological and social environment (Umwelt), body control being an important, but non-exclusive, part of this duty. Therefore, any species' brain possesses more processing capacity than those required to process body functions. The idea of dividing the brain in one part dedicated to cognition and another to body control is not new and has been criticized on the basis that discrete anatomical division is unlikely (Deacon, 1990a). In fact, both parts are probably linked in many ways. As Barton (2012) phrased it: “the evolution of large brains was associated with the elaboration of sensory-motor mechanisms for the adaptive control of bodies in their environments.” It is nonetheless possible to distinguish between the two in formulating the hypotheses on the factors underlying brain evolution. Indeed, what are the non-somatic factors that could have played a role in the evolution of brain size and composition?

#### Longevity

The variable “body size,” often represented by body weight, is only an approximation of a size factor (not necessarily a good one, Harvey and Krebs, 1990; Burish et al., 2010). This “size factor” is actually much more than a simple morphological variable and involves the allometric scaling of many other lifestyle variables. Of particular interest here is the observation that big mammals live longer than smaller mammals (Speakman, 2005). Logically, *absolute* brain size correlates with longevity (Sacher, 1959, Table 2 of Allman et al., 1993). Merker (2004) proposed that the size of the neocortex is an adaptation for the long term storage of contextual information needed throughout a species' lifespan. This hypothesis is close to that of Allman et al. (1993) with the major difference that Merker's account concerns the *absolute* size of the brain (or more exactly, the neocortex) correlating with *absolute* lifespan, and not their *relative* values. The original hypothesis of Allman et al. (1993) can in fact be adapted without even modifying the authors' original words: “one of the important functions of the brain is to store information about resources in the environment so that the organism can survive occasional catastrophes by switching to alternative resources. The longer the life-span of the animal, the more likely it is to encounter severe crises during its lifetime. Thus, it might be expected that species with longer life-spans would have larger brains in order to sustain individuals through the more severe crises likely to occur in a longer life” (Allman et al., 1993). Under this view, the large neocortices of big bodied mammals are an obligatory feature that permits the long-term storage of their longer “personal history” (Merker, 2004). Interestingly, the neocortex enlargement is coupled with the cerebellum enlargement (Barton, 2002, see also Sultan, 2002; Herculano-Houzel, 2010; Smaers et al., 2011b). In fact, in addition to its role in motor control, the cerebellum plays a role in many other cognitive functions (Ramnani, 2006), and it has been suggested that these two structures operate together, under a cerebro-cerebellar system (Leiner et al., 1991). Imamizu et al. (2000) have found that, in humans, the cerebellum possesses an internal model of new tools after learning. Given that a bigger lifespan implies more situations of learning, the enlargement of the cerebellum is compatible with the view that a fraction of brain enlargement responds to the need for storing the quantity of knowledge that goes along with longer lifespan. However, the relevance of the storage hypothesis is likely to vary across taxa. For example, it might be less relevant in bats (chiroptera), who have both small brains and a relatively high longevity (e.g., Brunet-Rossinni and Austad, 2004).

#### Information processing capacity

Many hypotheses on the cognitive factors that play a role in the evolution of the size and the composition of brains have been proposed. These hypotheses are traditionally separated into two categories; ecological [the spatiotemporal mapping hypothesis, Clutton-Brock and Harvey (1980); the foraging hypothesis, Milton (1981), Gibson (1986); the technical intelligence hypothesis, Parker and Gibson (1977), Byrne (1997)] and social (the social intelligence hypothesis, Humphrey (1976), Byrne and Whiten (1989), Dunbar (1998); the relationship intelligence hypothesis, Emery et al. (2007) [but see Scheiber et al. (2008); the cultural intelligence hypothesis, van Schaik et al. (2012)], although the two are sometimes linked (Dunbar and Shultz, 2007). Sensory motor factors have also probably played a role in the evolution of cognitive capacities via, for example sensory control of skilled movements (Whishaw, 2003; Sultan and Glickstein, 2007) or sensory adaptations (Paulin, 1993; Barton, 1998, 2004). Indeed, as stated by Barrett (2011) “An adaptive fit between an organism and its environment can also be achieved through selection for a capacity that allows animals to continually update their knowledge of the world.” In addition, there are evidences that body size could influence a species' cognition and behavior (Dial et al., 2008). Charvet and Finlay (2012) have suggested that the longer developmental time needed to construct larger brains and the extended learning period associated with it should be viewed as a factor in brain evolution. Therefore, it is possible that the factors reviewed above act differently on altricial and precocial species.

Each ecological niche is characterized by a certain combination of these factors. The adaptative approach presented here postulate that the respective importance of these factors should be partly visible in evolution of the brain architecture. In fact, there is direct evidence that increasing cognitive capacities have been a driving factor in brain evolution among simians. First, Bush and Allman (2004) have shown that the frontal cortex scales with positive allometry relative to the rest of cortex in primates while it does not in carnivores. Second, Balsters et al. (2010) have measured the volumes of cerebellar lobules in structural MRI scans for capuchins *Cebus apella*, chimpanzees *Pan troglodytes* and humans and have found a tendency for the lobules related to prefrontal cortex to get relatively bigger into the simian's cerebellum as the brain gets bigger. Although a definitive conclusion would require an appropriate dataset, this impression is supported by the macaque monkey *Macaca mulatta* measure that fits between the ones of the capuchin and the chimpanzee, as predicted by its intermediate brain size. Interestingly, there are direct pathways between the neocortex and medulla that become increasingly important with brain size in simians (Striedter, 2005). This suggests that, in simians, selection for higher processing capacity is also linked to a better cognitive control of the body inside the environment (see also Wilson, 2002; Barton, 2012).

It is important to note, however, that developmental and functional constraints could limit the extent to which brain regions can be selected individually. The neocortex, in particular, is divided in areas devoted to a particular set of information (see Krubitzer and Seelke, 2012 for an overview of their evolution) and there is a correlation between neocortex size and the number of neocortical areas across mammals (Changizi and Shimojo, 2005; Striedter, 2005) due to the constraints of maintaining neuronal connection when neuron number increases (Ringo, 1991). As neocortex gets bigger, higher order cortical areas emerge from core fields (Rosa and Tweedale, 2005, see also Kirkcaldie and Kitchener, 2007), leading to a better treatment of neural information (Striedter, 2005, but see Kaas, 2000). It is possible that, due to developmental and functional constraints, selection for some functions supported by the neocortex has consequences on the whole structure [but see Welker and Seidenstein (1959) for a counterexample]. In addition, it is possible that, compared to smaller ones, bigger structures are better able to integrate new functions (via neural reuse for example, Anderson, 2010) without a correlated increase in size.

Although much more work is needed to unravel the mechanisms and factors responsible for the evolution of brain composition, the approach presented here strongly contrasts with the traditional (allometric) approach presented above, in particular by giving a direct adaptive value for the size of each brain region. Moreover, this approach undermines the hypothesis that variations in brain structure sizes are solely the consequence of their position on the prosomeric axes (Finlay et al., 2001) and will ultimately permit understanding of the full complexity of the developmental model (including the cerebellum “exception” of the “late equals large” model). Other factors, cognitive or not, are likely to be added on the list of factors influencing the evolution of the size and the composition of brains. For example, the hypothesis of “adaptive redundancy,” which postulates that in larger brains “memories are written into multiple circuits to protect against interference or injury” (Chittka and Niven, 2009) can be extended to the whole range of cognitive process. It is also possible that the robustness associated with larger brains is physiological (in terms of blood flow, glucose reserve, etc.). Also, under the view developed above, the evolution of brain structure sizes in taxon-cerebrotypes is concerted mainly because species are under similar selection pressures. Importantly, the concerted pattern hides a host of species-specific adaptations in brain composition that further adapts each species to its own environment (see Krebs et al., 1989; DeVoogd et al., 1993 for early references). However, most studies on this issue should be re-evaluated in regard of the points discussed here, in particular the importance of examining both the absolute and relative size of a brain region (see also section Brain Composition and Cognition). It follows from above that the variable “brain size” should therefore be used with caution; as it hides the real variables under selection, the structures (see also Healy and Rowe, 2007).

## Relative brain size

The preceding section has shown that body size is only one among many other variables influencing brain size. Therefore, although encephalization is a multidimensional variable (dependent on all the cognitive, environmental and lifestyle factors associated with brain and body size), the EQ reduces it to a bidirectional (brain/body) approach. The true meaning of such an approximation has yet to be defined; in particular in view of the interest researchers have for it (e.g., Lefebvre, 2012 for a recent review in primates). For example, which structures participate in the relative (compare to body size) variations in brain size? A relatively enlarged brain is thought to represent “selection for enhanced processing capacity” (“active growth” Aboitiz, 2001). If true, what fraction(s) of the processing capacities have been favored, or reduced (long-term storage, sensory motor, or association capabilities) with encephalization? How do variations in structure size correlate with lifestyle variables?

### Brain composition and encephalization

**Figure 5:**
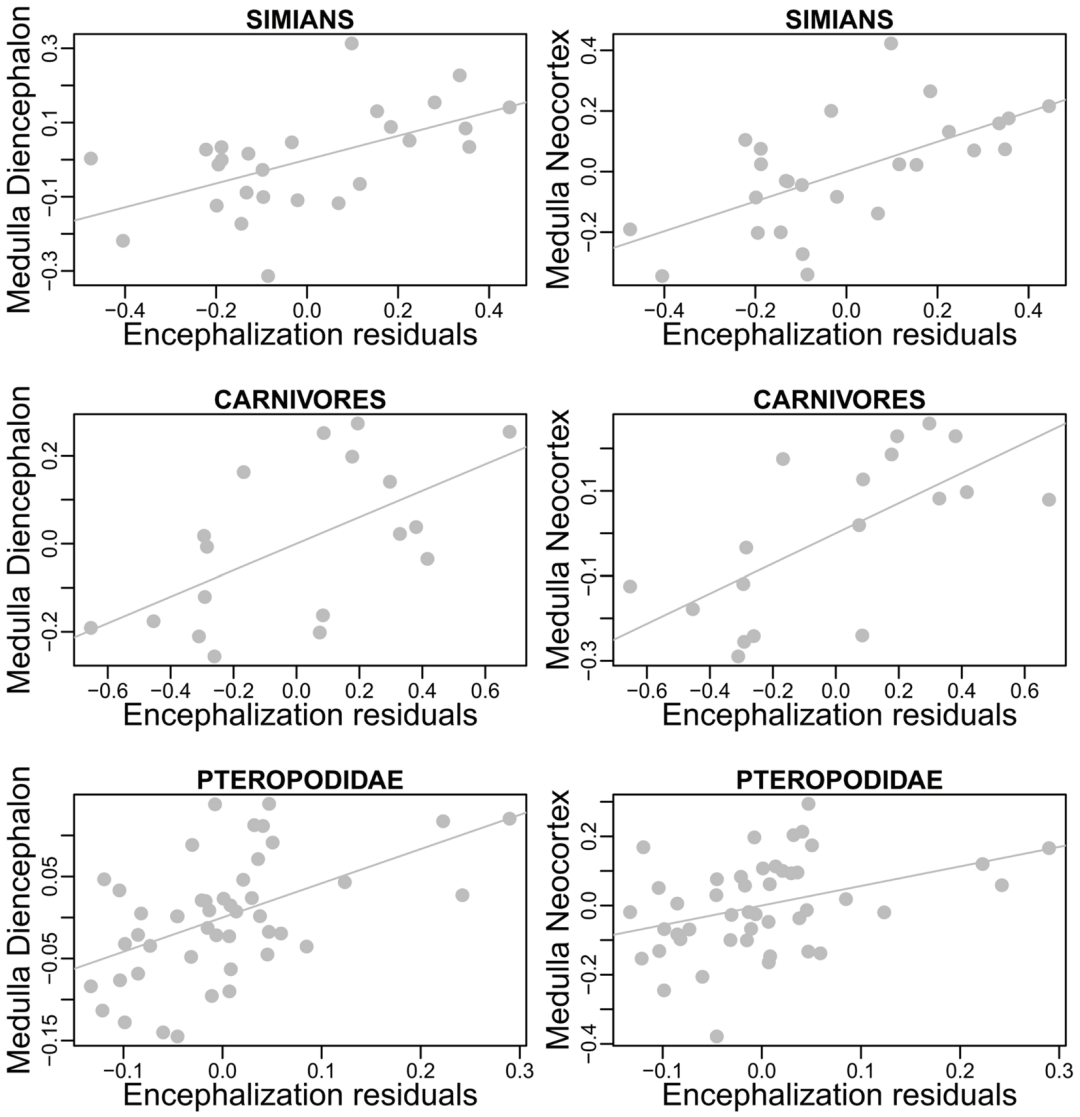
**Details from Table 2**. Residuals from regressions of diencephalon (left) and neocortex (right) size onto the size of the medulla regressed onto encephalization residuals.

The approach presented above predicts that species with relatively larger brains present specific modifications in the taxon-cerebrotype allometry reflecting the structures having been selected (see also Aboitiz, 1996). Early support for this hypothesis is found in Barton (1998) who reported that relative neocortex size correlates positively with the EQ. Crucially, the extent to which a structure participates in brain size variation depends on its relative and absolute size (see also Lefebvre et al., 2006). For example, in a hypothetical species with a 100 g brain, selection for the functions supported by a structure doubled the size of this structure from 5 to 10 g (changing its proportion from 5 to 9.5%). This species, although with a structure two folds bigger than the ancestral condition, still possesses a brain of almost the same size, 105 g. Because brain size has been almost unchanged, this adaptation is nearly invisible for analyses using absolute brain size or encephalization residuals. On the contrary, a 2-fold augmentation of a 50 g structure raises brain size from 100 to 150 g, one and a half initial brain size, whereas the structure's relative proportion goes from 50 to 66%, an augmentation of 1.3 only. Therefore, changes in the largest structures are the most likely to be detected by encephalization studies (and to cause changes in absolute brain size, see section Factors Underlying the Evolution of the Size and the Composition of Brains). To address this point, one can test the correlation between deviations from structure size allometry and encephalization residuals. The analysis, carried out in three relatively homogenous taxon-cerebrotypes: carnivores, simians, and the chiroptera's family Pteropodidae (see Willemet, 2012), suggests that the neocortex is not the only structure modified during encephalization, although larger datasets and phylogenetic methods are needed before definitive conclusions can be drawn (Table 2, Figure 5).

**Table 2:**
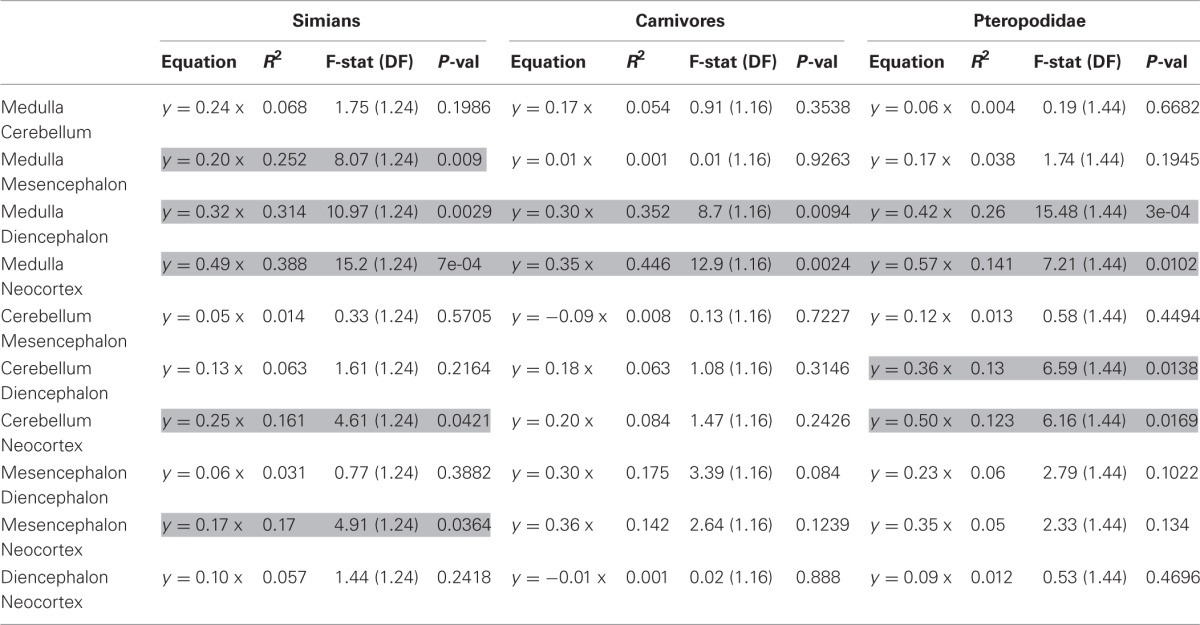
**Results of regression of residuals of the size of one structure against the size of another structure onto encephalization residuals**.

Significant regressions (P-values < 0.05) are indicated with cell fill in dark gray. Data for simians and Pteropodidae are from Stephan et al. (1981) and unpublished data from the same research group. Carnivore data for brain structure sizes are from Reep et al. (2007), and data for brain and body size are from Gittleman (1986) and Finarelli and Flynn (2009) [from Bininda-Emonds (2000) for pinniped data, female values]. The human species is not included because it is an outlier in encephalization residuals. For further details, see Figure 5.

The significant relationships between brain composition and relative brain size suggests that variations in relative brain size are at least partly due to similar pressures between species (it is likely, however, that the factors controlling variations in brain structures differ between these taxa). This finding, if confirmed, is interesting given that the method used here does not distinguish between changes in relative brain size that are due to enlargement of the brain and changes that are due to reduction of the body (but see Montgomery et al., 2010 and Smaers et al., 2012a). In particular, the correlations between structure deviations and encephalization residuals in simians could provide insight into some species characteristics, like the relatively large cerebellum of gorillas *Gorilla gorilla* (Rilling and Insel, 1998; Semendeferi and Damasio, 2000; Sherwood et al., 2004). Inversely, these results suggest that the human cerebrotype is closer to the simian cerebrotype than predicted by its EQ (not shown here), despite some claim about it having a relatively large neocortex (Rilling and Insel, 1999). This suggests that there has been selection toward a relatively large cerebellum in modern humans, as also suggested by the reciprocal evolution of the cerebellum and neocortex during human evolution (Weaver, 2005). These results further support the approach presented in section Factors Underlying the Evolution of the Size and the Composition of Brains and suggest that the interpretation that relative brain size is a factor of brain evolution (e.g., Smaers and Soligo, 2013) ought to be re-evaluated and considered to be a consequence of brain evolution. How each structure reacts to selection is difficult to interpret from this analysis because the reference used to determine the variations is the relative size of the brain itself. An alternative to the use of brain and body size, and one possible way to evaluate the quantity of brain relative to the somatic factor could be to use the spinal cord as a predictor (see also Burish et al., 2010 and Herculano-Houzel, 2011a). Deviations from structure size/spinal cord allometry could be a better way to measure the selection process of brain structures than deviations from brain/body allometry because it would allow the detections of changes in all brain structures, irrespective of their size. Unfortunately, published data on the spinal cord is scarce (MacLarnon, 1996; Burish et al., 2010). Finally, although Herculano-Houzel (2007) has suggested that neuron number in brain structures is independent of the EQ in rodents, larger datasets are needed to examine whether the selective pressures linked to the EQ produce distinct changes in structure composition.

### Cognitive factors

It follows from the above that the selective pressures responsible for a brain's relative size should in fact mainly target the functions supported by the neocortex (and to a lesser extent, the cerebellum) and therefore, affect species' processing capacities. It is unclear; however, which part(s) of the processing capacities (cognitive, sensory-motor, information storage) is/are favored (or reduced) by variations in encephalization.

The storage hypothesis is grounded on neocortical organization (Merker, 2004), so that any increase in neocortex size should lead to an increase in brain storage capacity, even without direct selection for it. Indeed, relative brain size correlates with relative lifespan in primates (Allman et al., 1993; Barrickman et al., 2008). As the precise architecture of the cortico-cerebellar system and the mechanisms underlying information storage are uncovered, it will be possible to test this hypothesis more precisely.

Selection toward sensory-motor capacities could be one of the factors underlying changes in relative brain size. As discussed in section Non-somatic Factors, larger neocortex size could automatically lead to finer sensory motor representation, again even without direct selection for it. For some species it is also possible that larger brains have been selected specifically for increasing sensory-motor capacities in one or several modalities. For example, Barton (1998) showed a correlation between relative brain size and the size of the parvocellular pathway of the lateral geniculate nucleus (a visual center of the thalamus involved in the analysis of fine detail and color) in primates (see also Barton, 2004). Similarly, the relative amount of visual input correlates with relative brain size in primates (Kirk, 2006, see also Garamszegi et al., 2002). It is unclear, however, the extent to which selection for superior sensory-motor abilities can lead to increase in absolute (and therefore, relative) brain size. For example, although the North American raccoon *Procyon lotor* has a very large forepaw cortex that matches the extensive use of its hands (Welker and Seidenstein, 1959), there is no evidence that such a change can lead to a significant increase in brain size.

The most studied factor of encephalization is the cognitive buffer hypothesis, in which a relatively enlarged brain “facilitates the construction of behavioral responses to unusual, novel or complex socioecological challenges” (Sol, 2009). As for the storage and sensory-motor hypotheses, the cognitive buffer hypothesis could partly be a necessary consequence of increased neocortex size. Unfortunately, support for the cognitive buffer hypothesis often comes from studies that have mixed several taxa, and thus, several taxon-cerebrotypes [reviewed in Healy and Rowe (2007)]. Furthermore, for reasons that follow the discussion above, and detailed in the next section, the EQ cannot be systematically linked with *absolute* measures of cognitive abilities. If the encephalization process were associated with increased cognitive capacities, then a positively encephalized brain should have greater cognitive capacities than a similarly sized brain with neutral encephalization. In primates, the Pearson correlation test between residuals from the regression of an estimate of general cognitive ability onto the size of the brain on one side, onto encephalization residuals calls for further studies (*t* = 1.9854, *df* = 21, *p*-value = 0.06032, cor = 0.4, psychological data from Deaner et al., 2006). Notwithstanding the fact that the precision of the data is critically small and that many factors other than structure size control cognitive abilities (see sections Brain and Cognition; Species Differences in Cognition and Behavior), one of the reasons for this mixed result is the possibility that the size constraint has selected for mechanisms that increase brain efficiency in species with relatively small brains (Strasser and Burkart, 2012). A related hypothesis is that species that have undergone a decrease in brain size (see Montgomery et al., 2010) have kept some of the anatomical or physiological mechanisms evolved by their ancestor to counter the constraints of brain size (this could be an important factor in understanding the technology of the small brained *Homo floresiensis*, see for example Morwood et al., 2005). In all cases, searching for encephalization correlates is probably rendered difficult because many factors potentially influence brain structure sizes; to some extent in a species-specific way.

### Correlation between brain size and other lifestyle and environmental variables

While having a large brain is cognitively advantageous over a smaller one, the evolution of brain size has not been a one-way process (Niven, 2005; Safi et al., 2005; Montgomery et al., 2010; Smaers et al., 2012a). Many hypotheses on this issue suggest some kind of trade-off between brain (energetically expensive Aiello and Wheeler, 1995) and physiological or lifestyle variables. These hypotheses include metabolic constraints (e.g., Martin, 1981; Isler and van Schaik, 2006, but see Jones and MacLarnon, 2004), maternal investment (e.g., Martin, 1996; Finarelli, 2010; Barton and Capellini, 2011), neonatal maturity (Weisbecker and Goswami, 2011) and energy trade-off hypotheses [e.g., Aiello and Wheeler, 1995 (but see Hladik et al., 1999); Navarrete et al., 2011]. How these factors could influence brain size is discussed in their respective paper (see also section Methodological Issues).

However, the notion of “trade-off” to which these studies often refer is particularly difficult to test in a multidimensional world, as it entails more than a simple negative correlation between two variables. For example, although Pitnick et al. (2006) have found a correlation between relative brain size and mating system, adding only one variable to the analysis (morphological adaptation to foraging strategy) changes the allure of the results, with no correlation between testes mass and relative brain size (Dechmann and Safi, 2009, see also Lemaître et al., 2009). By increasing (or decreasing) its quantity of brain, a species changes the exploitation of its environment as well as the lifestyle variables that are associated with these changes. Therefore, although the notion of ecological constraints on brain size is particularly compelling (e.g., Winkler et al., 2004), the notion of “trade-off” should be called only when sufficient evidence supports it. Kotrschal et al. (2013) recently studied the possible trade-off between brain and gut size and offspring number. Although these results have been reported as “compelling experimental evidence for the cost of increased brain size,” the “trade-off” discussed in the expensive tissue hypothesis results from a limited amount of resources for which brain and gut evolutionary “compete.” In contrast, nutrients were abundant in Kotrschal et al. (2013) experiment, so the notion of cost has still to be precisely defined (see also Warren and Iglesias, 2012). Moreover, as discussed below, results from intra-species studies cannot be systematically transferred to the species level. The complex and interrelated relationships between all the cognitive, environmental and lifestyle variables, and brain and body size and composition constitute a global evolutionary strategy. This concept of strategy rectifies the notion behind the trade-off approach that all species hypothetically tend to have a bigger brain, but that only some can offer one because of the costs that it entails. Furthermore, it removes the apparent paradox that brain size could have cognitive implications while being determined by lifestyle constraints (Deacon, 1990a).

## Brain and cognition

### Brain size and cognition

In primates, by far the most studied mammalian taxon, several authors have recently suggested that absolute brain size could best explain species differences in cognitive abilities (Gibson, 2002; Deaner et al., 2007; Lee, 2007; see also Dunbar, 1992, but see Amici et al., 2010; Schmitt et al., 2012 and section From Cognition to Behavior: The Role of “Mentality”). This result was so counterintuitive that it is emphasized in the title of one of these influential papers “Overall Brain Size, and Not Encephalization Quotient, Best Predicts Cognitive Ability across Non-Human Primates” (Deaner et al., 2007). However, despite this new gain of interest toward absolute brain size (e.g., Marino, 2006), a coherent framework is still lacking.

**Figure 6:**
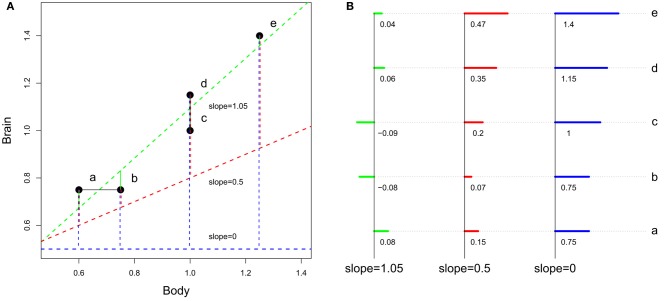
**Differences between encephalization quotient and absolute size in estimating species cognitive capacities. (A)** Representation of the slopes. **(B)** Detail of the residuals (the scale is not respected between the 3 cases). At slope equals 1.05 (slope obtained by a regression of hypothetical brain and body size), encephalization quotient is insensitive to brain size. At slope between 0 and 1, encephalization quotient is sensitive both to absolute and relative brain size. At slope equals 0, encephalization quotient equals absolute brain size. Although the preceding section has shown that brain size does not directly depend on body size, residuals have been computed using linear regression, the most used method for computing encephalization residuals (but see Warton et al., 2006 and O'Connor et al., 2007 for discussions on the regression methods used in comparative biology).

As discussed in section Factors Underlying the Evolution of the Size and the Composition of Brains, absolute brain size (through the scaling of absolute features like the number of neurons and cortical areas and the structures' relative sizes) is linked to species differences in processing capacity. Yet, the somatic factor hypothesis suggests that the “amount” of processing capacity dedicated to somatic factors also increases with body size. Therefore, for two species of similar brain size, but of different body sizes, the cognitive advantage should be for the smallest bodied species. However, as illustrated in Figure 6, none of these two variables, absolute brain size or EQ, can potentially take the other into account.

**Figure 7:**
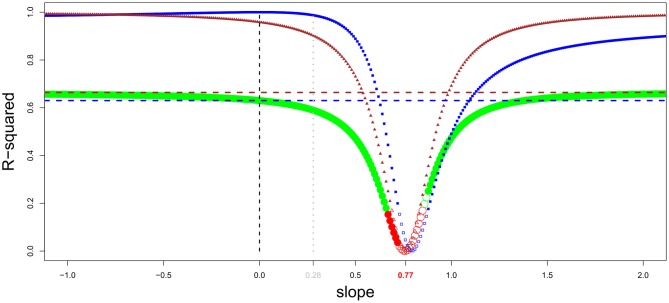
**General intelligence scores and encephalization slope in primates (brain and body data from Kirk, 2006, general intelligence scores from Deaner et al., 2006)**. Residuals from regression of brain onto body masses have been obtained by varying the encephalization slope value from −1 to 2 (step = 0.01). For every given slope, a linear regression between intelligence scores onto brain/body residuals has been carried out. The resulting *r*-squared is indicated by green dots when the regression is significant, and by red dots when it is not. In addition, the dots are filled when residuals correlate with brain size, and empty when not. The correlation between brain/body residuals and intelligence score lessens when the slope value approaches the value from a linear regression of brain onto body size (0.77). The *r*-squared from a regression between brain/body residuals onto body and brain size are shown by triangle and square lines, respectively; with filled symbols for significant correlations. The horizontal dotted line represents the percentage of variance in intelligence scores explained by absolute body (brown) and brain (blue) size. Using a slope equals to 0.28 (Williams, 2002; Alba, 2010) is unwarranted.

More generally, it would be interesting to have, inside a taxon-cerebrotype, an approximation of the fraction of brain size that responded to the extended somatic factor (that is, all the factors associated with body size) and the fraction dedicated to cognitive function. One possible method is to observe the correlation between encephalization residuals and a general measure of cognitive ability when the value of the encephalization slope (and therefore the effect of body size on brain size) varies. Figure 7 represents this analysis carried out in primates, the only taxon (although significant differences between major primate impose finer analyses, see Isler et al., 2008; Willemet, 2012) for which a dataset vaguely corresponding to such a measure exists (Deaner et al., 2006).

Figure 7 corroborate earlier findings that brain size better predicts species cognitive ability than does EQ in primates and suggests that the extended somatic factor have played only a limited role on the evolution of brain size in primates. This is in accordance with the evidences presented in section Information Processing Capacity, which suggest that selection for large cognitive abilities played a major role in simian brain evolution. The results of this analysis appear to be counterintuitive; however, as body size appears to better predict cognitive capacities than brain size itself [as in Deaner et al. (2007)]. A part of this result is probably due to species-specific adaptations. Moreover, Deaner et al.'s (2006) data on species differences in cognitive abilities are neither accurate nor comprehensive. With quality data, the allure of this analysis in other taxa could be particularly interesting for understanding the relation between brain size (absolute and relative) and cognition. Indeed, it is possible that, in other taxa, most of the variation in brain size has resulted from selection to the extended somatic factor and that only species that had selection for more cognitive abilities have increased the size of the brain regions associated with it. In these taxa, the EQ (or residuals from a slope close to it) should better predict cognitive abilities than absolute brain size (although even in that case, the processing capacity added should differ between species, see Herculano-Houzel, 2007 for an explanation based on neuron numbers).

At the cellular level, Herculano-Houzel (2007) has first proposed that “the total number neurons in excess of the expected from body size in each species” would be an indicator of species cognitive abilities. However, such a method is subject to the same flaws as the EQ method discussed above. Due to their proportional scaling with the number of spinal cord neurons, the number of neurons “expected from body size” has then been approximated by the number of neurons in structures others than neocortex and cerebellum (Rest of Brain, RoB, Herculano-Houzel, 2011a). Because RoB neuron number is low compared to total brain neurons, the author went on to suggest that, in mammals, “the cognitive abilities of a species might be simply a function of its total number of brain neurons” (Herculano-Houzel, 2011a). However, neurons in the cortico-cerebellar system in particular are not equally distributed among taxa (e.g., Bush and Allman, 2004) and areas (e.g., Cahalane et al., 2012) and species differ in neuronal connectivity (DeFelipe et al., 2002), so that two brains with a similar number of neurons should differ in their processing capacity. Therefore, studies at the neuronal level will need to be particularly detailed, in the number of species, structures and cell types scanned, to reveal their whole potential.

In conclusion, the variables “brain” and “cognition” both represent a set of variables which are particularly complex (see also section Species Differences in Cognition and Behavior), so that any attempt to link these two “meta variables” will always be a rough approximation of the relationship between the variables that constitute them. As stated by Barton (2012), “the search for a single ideal comparative brain measure that captures the neural basis of cognitive evolution is likely to be more obfuscatory than illuminating, because different selection pressures have acted on different neural systems at different times.” Therefore, far from being “fruitless” (Jerison, 1985), looking at finer correlates of cognitive abilities is a fundamental issue in comparative neuroscience.

### Brain composition and cognition

As pointed out by Striedter (2005) through the example of the small but crucial suprachiasmatic nucleus (that controls circadian rhythms), the importance of a structure in the brain network or in a species' life are two different things. However, absolute features of a brain structure, such as its size and the number of neurons and synapses can theoretically have an impact on the structure's computational power (Striedter, 2005). For example, the particularly sensitive and agile hands of raccoons are linked with a particularly large cortical representation of the forepaws (Welker and Seidenstein, 1959). Importantly, however, whether a bigger structure generates higher processing capacity is true only when the advantages of being larger compensate for the functional constraints on conduction time and neuronal connectivity for example. Therefore, the relation between structure size and processing capacities is not necessarily linear and should depend on the intimate composition of a structure determined by the taxon-cerebrotype characteristics (such as the scaling of cell number in structures, Herculano-Houzel, 2011b). The relative size of brain structure also plays a role in the structure's importance inside the brain network. The principle of “large equals well-connected” (Striedter, 2005 after Deacon, 1990b) states that the larger a structure becomes in evolution, the more its neurons invade the other regions, accenting the region importance in the brain network. It is likely, however, that evolutionary mechanisms (like favoring intra-structure neuronal connection) limit this “invasion” when it is not adaptive.

Because brain structures or regions rarely support only one function, and because apparently similar behaviors can have different underlying cognitive mechanisms, understanding how brain composition influences cognitive abilities is complex. For example, the mechanisms for food hoarding behavior (one of the most studied behavioral traits) vary from simple retrieval mechanisms (Brodin, 2005; Smulders et al., 2010) to probably multi-dimensional maps in which the age and content of the cache is remembered (Clayton and Dickinson, 1998). Moreover, and in addition to the fact that studies often mix various taxa, a number of methodological issues have been highlighted in previous papers (e.g., Bolhuis and Macphail, 2001; Healy and Rowe, 2007; Roth et al., 2010 see also next subpart). Furthermore, the size of a structure is not the only parameter that accounts for its function (Roth et al., 2010), forcing neuroecologists to examine different levels of analysis (Ball et al., 2002; Pollen and Hofmann, 2008). By their correlative nature, comparative analyses are insufficient for studying the mechanisms underlying behaviors so that a back and forth paradigm between experimental and comparative analyses is probably needed to study the neuronal correlates of complex behaviors (Pollen and Hofmann, 2008; Smulders et al., 2010) and to eventually draw a evolutionary framework on these characters (MacLean et al., 2012).

### Methodological issues

In addition to the points raised above, three issues in particular affect comparative studies on brain, cognition, and behavior.

1. The strength of a hypothesis directly depends on the strength of the data on which it is based. Yet, datasets are often too small, making results difficult to interpret. Twenty years ago, Jerison already complained about “how much longer we will have only Stephan and his colleagues for appropriately large samples of measures of the brain” (Jerison, 1993). Except for the contribution of Reep et al. (2007) and some sporadic additions of new mammalian species, the number of species for which brain composition is known has steadied, as have intra-species measurements. This is problematic, since the acuity of the determination of cerebrotype characteristics directly depends on the number of species for which data are available (see also Yopak, 2012). Indeed, while primates are the mammalian order for which there is the largest amount of anatomical and lifestyle data (e.g., Dominy et al., 2004; Striedter, 2005; Preuss, 2007; Barrickman et al., 2008; Herculano-Houzel and Kaas, 2011), data on brain structure is available for less than 25 percent of species (estimated at around 200, Purvis, 1995). Similar conclusions apply to birds, for which high quality data is only available for a limited number of species (Boire and Baron, 1994; Iwaniuk et al., 2004). Fortunately, imaging studies, beside ethical and free from shrinkage problems, can now be used for getting brain measurements (e.g., Semendeferi and Damasio, 2000; Sherwood et al., 2004). After decades of testing, the most complete data on comparative cognition in mammals concerns the primate taxon (Deaner et al., 2006; Reader et al., 2011). However, primate data is incomplete in the number of tests done by each species, requiring complex statistics to compensate for the lack of data (Johnson et al., 2002; Deaner et al., 2006) while still being at the genus level. Data from the wild (e.g., Reader et al., 2011 in primates) represents an important source for mapping species differences in cognitive abilities (Kamil, 1987; Byrne and Bates, 2011). Indeed, ethical methods of data acquisition are developed to test wild or semi-wild animals (e.g., Fagot and Paleressompoulle, 2009; Woods and Hare, 2010; Marino and Frohoff, 2011; Gazes et al., 2013; Healy and Hurly, 2013). Finally, although a few model species in the laboratory have been extensively studied, most of them have been raised for decades in artificially poor environments that potentially affect normal brain functioning (Würbel, 2001 and see section Intra-specific Analyses). Whether it is relative to brain composition or cognitive abilities, increased collaborations, and data sharing are the keys to improving this point (Tomasello and Call, 2011; MacLean et al., 2012).
2. Because they compare species with shared evolutionary history, comparative studies do not fill the condition necessary for most statistical methods; the independence of data points (Felsenstein, 1985). The use of sophisticated comparative methods (MacLean et al., 2012) associated with high quality phylogenetic trees (e.g., Arnold et al., 2010) is a necessary step to improve our understanding of the evolution of brain, cognition, and behavior.
3. Whether a correlation is significant or not is only a statistical description of the data, and is insufficient to state that two biological variables co-vary. In other words, *p*-values and regression coefficients are not enough and two questions should systematically be asked in regression analyses. The first is; can the regression predict the value of the variable of interest in a *biologically significant way*? The second is; can we explain the potential extreme values? Only when these two criteria satisfied are the regression analyses strong enough to serve as basis for other studies. In the other cases, future studies should clarify the relationships between the variables. Importantly, because of the uncertainty existing in variable relationships and the often small datasets available, results from multiple regressions (or related methods) should be interpreted particularly cautiously.

Unless proven otherwise, these issues, and those discussed above, should necessarily have adverse consequences for each paper in which they are found (including some papers reviewed here), so that a number of published works will probably need to be reanalysed as soon as better data is available. Consequently, readers should keep the following biases in mind when referring to previous papers or when designing future research.

## Species differences in cognition and behavior

### Cognition

Deacon (1990a) remarked that “no one would consider ranking such mammals as dolphins, rabbits, moles, horses, bats, and gibbons according to some linear scale of locomotor efficiency, capacity, or competence.” Similarly, such an observation could be true for cognitive abilities. However, in the case of locomotor efficiency, one can measure species speed or endurance for instance, and this makes sense, for example, when considering prey/predator interaction. Likewise in the case of cognition, one can consider ways to approximate the ability to resolve problems or the ability to have a complex mental representation of the world. Indeed, Andrews (2011), qualifies the current period as “a kind of golden era when it comes to animal cognition research.” Considering the number of papers published or the number of subjects tackled, Andrews's observation is certainly right. However, scientists still have a limited understanding about the very nature of cognition and its variation between species. In fact, our understanding of the cognitive capacities of non-human and human animals is continuously remodeled by new experiments. Moreover, in a number of papers many species are tested for the presence/absence of cognitive abilities. Such a binary approach can potentially hide quantitative differences between species (Wright, 2010). But maybe the biggest difficulty is that a deep understanding of cognition cannot be achieved without taking into account the brain mechanisms underlying it. This is evidently true for detailed analyses at a species level, but it also holds for comparative studies.

For example, Deaner et al. (2006) found that various cognitive measures have strong positive inter-correlations in primates, thereby supporting the hypothesis that “primate taxa differ in some kind of domain-general ability” (see also Reader et al., 2011). However, the presence of such a “general intelligence” factor in primates could be the consequence of the concerted scaling of the brain architecture supporting primates' cognitive abilities (see section Factors Underlying the Evolution of the Size and the Composition of Brains), instead of being an inherent property of cognition. Similarly, Lefebvre and Bolhuis (2003) noted that, in birds, “the negative correlation [between innovation rate and food storing] is consistent with the idea that there has been a trade-off between the demands of storing and innovation” (brackets added), supporting the view of “limited modularity in animal cognition.” However, roughly speaking, if food storing depends closely on the hippocampus and learning and innovation on other nuclei, then the limited modularity of cognitive capacities directly reflects the architecture of the brain (suggesting that different cognitive strategies have been selected). If follows from these two points that understanding the modularity of cognition requires the study of the neurological bases of cognitive abilities. For example, while chimpanzee and bonobo *Pan paniscus* are phylogenetically very close (Fischer et al., 2011), the significant differences in their behavior (Doran et al., 2002; Hare, 2009) and cognitive abilities (Herrmann et al., 2010) correlate with slight differences in their brain architecture (Rilling et al., 2012, see also Hopkins et al., 2009).

### From cognition to behavior: the role of “mentality”

There are species-specific ways to react to a noise, a conspecific, another species, etc., that go beyond species respective cognitive capacities and that determine, alongside the perceptual abilities proper to each species, what kind of information, through all the information available in the environment, is analysed, and how. Although much less studied than species differences in cognitive capacities, the terms “temperament” (Réale et al., 2007) and “behavioral syndrome” (Sih et al., 2004) have sometimes been proposed to account for this aspect of animal behavior at the individual, population, species, and even group of species levels. This lack of specificity is potentially problematic in a neuroecological approach, since the neurological bases underlying behavioral differences at the species and individual levels possibly differ. For this reason, the term “mentality” has been used here to describe the species-specific way to analyse and react to their environment.

The concept of mentality as defined here encompasses the array of behavioral differences that are not directly due to species differences in cognitive abilities; such as patience (Stevens et al., 2005; Rosati et al., 2007; Addessi et al., 2011; Pelé et al., 2011) and inhibitory control (Amici et al., 2008), differences in risk preference (Heilbronner et al., 2008), neophilia and exploration (Parker, 1974; Mettke-Hofmann et al., 2002, 2005; Bergman and Kitchen, 2009) among others. Such differences between species could result from large or small changes in the pattern of brain structure (e.g., Rilling et al., 2012) or neuropeptides (e.g., Young, 1999; Lim et al., 2004; Goodson and Kingsbury, 2011) for example, controlling how species collect and process information about their environment (see also Lotem and Halpern, 2012). Because of the homogeneity of brains inside a taxon-cerebrotype, species generally act in a closer way than compared to species from other taxon-cerebrotypes. Although this issue has still to be studied thoroughly (see for example Auersperg et al., 2012), mentality differences between species could have profound effects on species apparent cognitive abilities (e.g., Greenberg and Mettke-Hofmann, 2001; Greenberg, 2003; Hare, 2009; Byrne and Bates, 2010). Indeed, (Amici et al., 2012) found that in seven primate species, performances in several cognitive tasks correlate with certain properties of their social system for which mentality plays an important role. Mapping species differences in mentality along with differences in cognitive abilities is therefore necessary to understand species differences in behavior.

## Intra-specific analyses

### Brain/body and brain structures scaling

**Figure 8:**
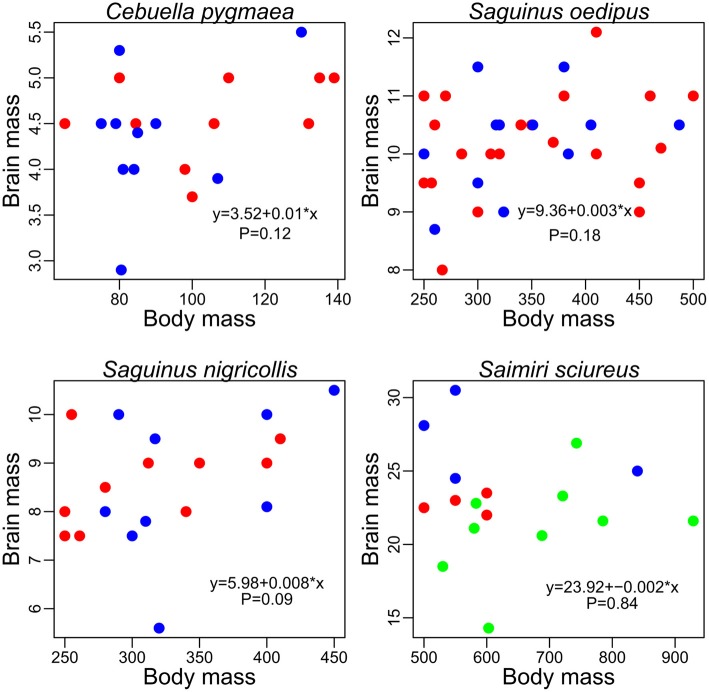
**Brain and body mass (grams) in pygmy marmoset (*Cebuella pygmaea*) two species of tamarin (*Saguinus nigricollis* and *Saguinus oedipus*) and in squirrel monkeys (*Saimiri sciureus*), data from Hartwig et al. (2011)**. Females, red; males, blue; unknown sex, green.

Some individual minks *Mustela vison*, have brains up to 40% larger than others [mean brain around 9 g, data from Kruska (1996)]. Likewise, differences between the smallest and largest brains reaches 800 g in humans (mean brain around 1300 g, Holloway, 1980) and more than 2000 g in elephants *Loxodonta africana* (mean brain size roughly equal to 5000 g, Shoshani et al., 2006). Because large individuals need larger organs, muscles or bones, it could be expected that a part of the variance in brain size is explained by body size. In minks for example, there is a strong correlation between individual brain and body sizes [cor = 0.97, data from Kruska (1996) range body weight: 510–1272 g]. However, in other species such as primates species (see Figure 8, see also Heymsfield et al., 2012) and at least some bird species (e.g., Møller, 2010) the relationship between brain and body mass is weak or absent. In fact, how body size influences brain size inside a species is unknown and has still to be examined with a large and systematic comparative dataset (see also Holloway, 1980). Species with important sexual dimorphism, such as pinnipeds (Bininda-Emonds, 2000; Fitzpatrick et al., 2012) would be particularly interesting (see also Falk et al., 1999). Also, the process of domestication (Belyaev, 1979; Price, 1999; Trut et al., 2009) is of particular interest here, because of the variability between brain and body size among different breeds [review by Kruska (2005)]. Such studies could ultimately clarify the degree to which body size variations influence brain size (for example via constraints on skull size, e.g., Morriss-Kay and Wilkie, 2005). There has been intense debate concerning whether body size should be accounted for when considering brain size differences between human groups (Peters et al., 1998) or during evolutionary history of the human species (e.g., Pilbeam and Gould, 1974). Ultimately, such issues should be studied by analysing inter-individual variation in brain structure sizes.

**Figure 9:**
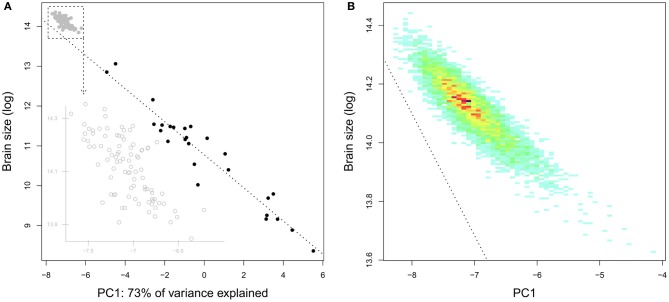
**(A)** Brain size as a function of the species position on the first component of a principal component analysis of simians' brain structure proportions (the structures included are medulla, cerebellum, mesencephalon, diencephalon, striatum, septum, olfactory cortex, hippocampus, subicular cortex, neocortex). The position of the human individuals (gray) has been predicted by the PCA done with simian species (human species excluded). **(B)** Density plot based on the position on the precedent first component of 5000 simulated brains constructed by taking 5000 random values for every structure with a normal distribution and a mean and standard deviation similar to the real dataset. The dashed line indicates the regression slope for simian brain size as a function of species position on the principal component analysis.

Finlay et al. (2011) have studied the inter-individual variations of brain structure sizes in minks, pigs, and laboratory mice, and concluded that “the pattern of individual variation in brain component structure […] is very similar to variation across species in the same components, at a reduced scale.” Although the inclusion of domestic individuals is potentially problematic (see below), this hypothesis is apparently supported by a study using brain data from 90 young adult humans (Charvet et al., 2013) which suggests that the same (developmental) mechanisms could be responsible for both within and between species variations in brain anatomy. As stated in sections Comparative Brain Studies in Birds and Mammals and Comparing Taxon and Species Cerebrotypes, the patterns of brain variation at the mammal level to which these studies refer to are relatively uninformative (see also Willemet, 2012). Since the range of variation between human brains is larger than the range of variation in all the simian species altogether, such a large dataset is particularly interesting for understanding the intra-species variation of brain composition. Indeed, a principal component analysis on brain structure proportion shows that the pattern of variation of human brain composition seems in continuity with the pattern of variation of simian brain composition (Figure 9A).

**Figure 10:**
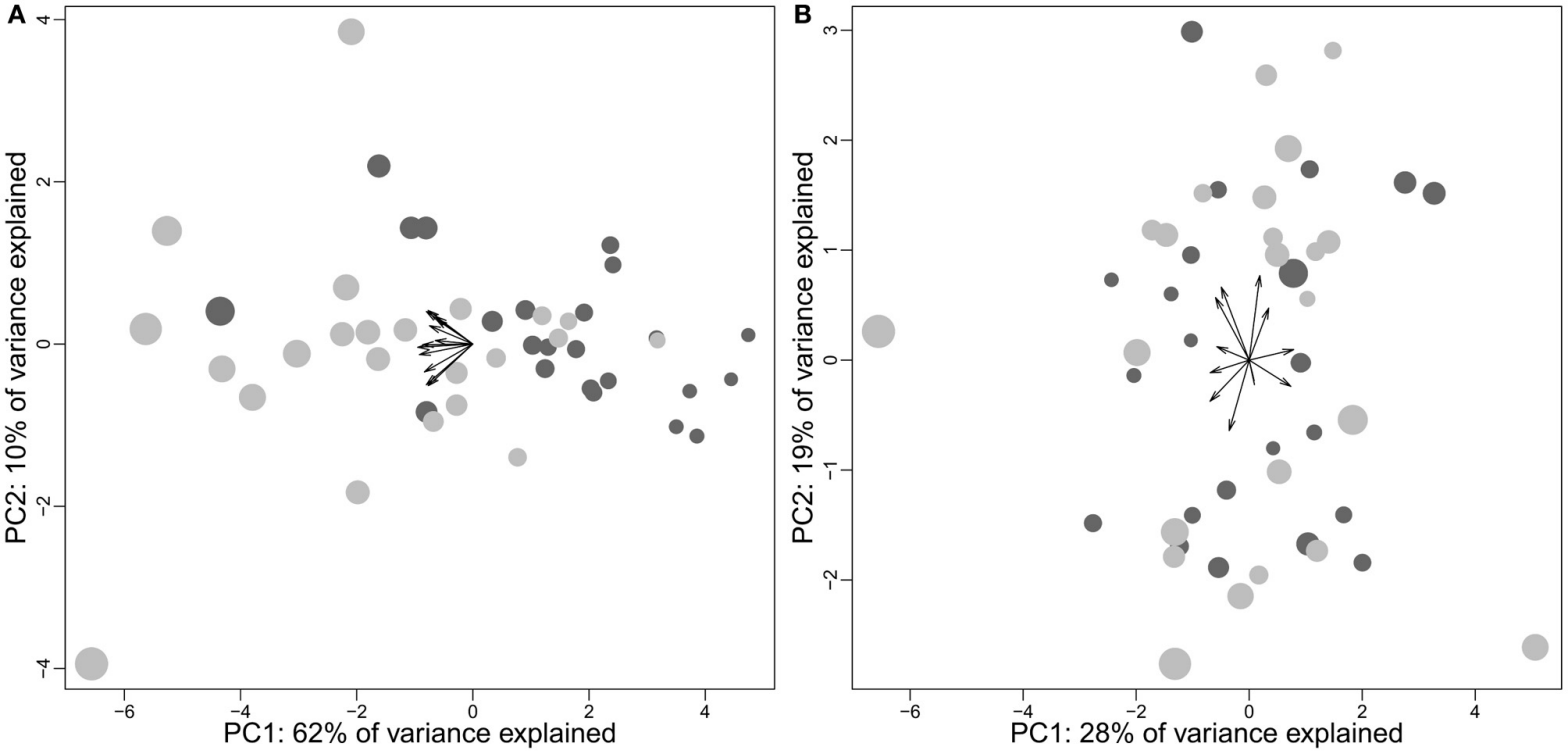
**Principal component analyses of cortex subparts in humans using absolute sizes (A) and structure proportions (B)**. Data come from Allen et al. (2002) and consist in the volumes of 12 cortical regions (frontal, temporal, parietal, occipital, cingulate, insula on both hemispheres). Females, dark gray; males, light gray. **(A)** Correlation between neocortex size and position on PC1: cor = −0.99, *t* = −42.3528, *df* = 44, *p*-value < 2.2e-16; **(B)** Correlation between neocortex size and position on PC1: cor = −0.02, *t* = −0.165, *df* = 44, *p*-value = 0.8697. The arrows represent the structure loadings on the two firsts principal components.

The resemblance could be superficial, however. In particular, a large part of this result (and of those of Charvet et al., 2013) could be due to the phenomenon described in section Relative Brain Size; large size differences between human brains are likely to be due to differences in the biggest structures: the neocortex and to a lesser degree, the cerebellum; thereby resembling species variation across species. Indeed, repeating the analysis on simulated brains shows a similar pattern (Figure 9B). Furthermore, the hyperscaling found for primate frontal cortex (Bush and Allman, 2004) is not found in the human sample. In fact, it seems that there is no predictable variation of cortical composition in human (Figure 10).

These results suggest that the mechanisms underlying inter-individual variations in brain structure sizes might be different from those having been selected at the species level. Understanding the genetic basis of some particular brain conformation and in particular in humans, in microcephaly (e.g., Mahmood et al., 2011) and rare developmental disease (e.g., Manzini and Walsh, 2011; Netchine et al., 2011), could probably shed light on some of the mechanisms controlling brain size and composition inside a species (and to some extent between species, e.g., Gilbert et al., 2005; Molnár et al., 2006). Also interesting are the seasonal variations of brain structure sizes in some birds and mammals (Yaskin, 1994; Jacobs, 1996; Tramontin and Brenowitz, 2000; Sherry and Hoshooley, 2010). Evo-devo researchers have already described some of the mechanisms that have been selected during brain evolution [review in Charvet and Striedter (2011), see also Lipp and Wolfer (2002) and Katz (2011) for discussion on the evolution of complex nervous system]. Accordingly, it has been shown that the size of brain structures is heritable (Airey et al., 2000; Thompson et al., 2002; Fears et al., 2009) and controlled by independent loci (Hager et al., 2012). In controlled conditions, selection for a particular behavior (high voluntary wheel running) has been associated with larger midbrains in domestic mice (Kolb et al., 2013). Interestingly, sex-differences in brain architecture in primate species suggest that female and male brains could be under different sets of selection pressures (Lindenfors et al., 2007; Smaers et al., 2012b). For this reason, sex should be accounted for in studies on the selection pressures underlying the evolution of the brain.

Domestication represents a particularly valuable resource for understanding how brain structures can be selected at the species level (see Kruska, 2005 for a review and Gleich et al., 2000 and Rehkämper et al., 2003, 2008 for recent contributions). What emerged from this body of work are profound differences in brain composition between domesticated and wild forms [review in Kruska (1988, 2005, 2007)]. However, the number of individuals per species and per condition (wild vs. domestic) is often very small (typically less than 4–6). Moreover, the method used in most studies [detailed in Ebinger (1974)] confounds the effect of brain reduction (or body size augmentation) as well as absolute and relative changes in structure volume. This makes the results difficult to interpret. For example, while mean neocortex size is larger in ranch individuals than in wild minks (4802 vs. 4622 mm^3^), this method gives a 17.8% reduction of neocortex size from wild to ranch minks (Kruska, 1996). In spite of this, reiterating the analyses with a different method supports one of the original conclusions that in most species, the neocortex is the structure most affected by domestication (Finlay et al., 2011). Although the reasons for such changes are unknown, this suggests that some functions supported by the neocortex may have become unnecessary during domestication, or that some mechanisms permitting a decrease in brain structure size while preserving (at least some of) the functions have come along with domestication. In fact, is is possible that most of the neurological differences between domestic and wild individuals are to be found in factors other than size (e.g., Saetre et al., 2004).

### Inter-individual differences in cognition

In humans, where most studies have been conducted, both the genetic bases (Frank and Fossella, 2011; Green et al., 2012 but see Deary et al., 2009; Chabris et al., 2012) and neuroanatomical correlates of individual differences in cognitive abilities are still unclear. A significant amount of evidence suggests that in humans, the scores obtained in different cognitive tests correlate with each other, so that a global factor, called “g” for general intelligence, accounts for an important part of total variance (at least 40 percent, Deary et al., 2010). This suggests that cognitive abilities are not totally independent of each other, sharing (at least partly) a common mechanism (e.g., Ebisch et al., 2012 but see also Rabaglia et al., 2011). In fact, more and more evidence indicates that general cognitive abilities originate from a network of interconnected cortical areas (Deary et al., 2010). Indeed, it seems that intelligence correlates with brain size in humans (correlation around 0.3 McDaniel, 2005, but see Schoenemann et al., 2000). Absolute features such as the degree of girification (e.g., Germanaud et al., 2012) or neuron number (at least in the neocortex; Pakkenberg and Gundersen, 1997) could probably explain a part of this relationship. However, whether inter-individual variations in structure size and neuron number always correlate has still to be studied thoroughly (in the number of species, individual per species, and structures). For example, in 9 owl monkeys individuals *Aotus trivirgatus*, Collins et al. (2013) have found no significant correlation between the masses of several visual brain structures and neuron number. Inter-individual differences in cognitive abilities go beyond such a general factor. Indeed, human studies have shown that for every cognitive test, part of the unexplained variance “reflects the particular abilities involved in the test” (Deary et al., 2010). More exactly, particular cognitive abilities seem to be supported by localized brain areas (e.g., Johnson et al., 2008, see also Gläscher et al., 2010). While the understanding of the neurological bases of individual differences in cognitive abilities is still in its infancy, it is complicated by the role of environment in both brain composition and cognitive abilities (Mohammed et al., 2002; Simpson and Kelly, 2011), and by individual differences in perceptual abilities (Kanai and Rees, 2011).

The considerable work in humans contrasts with the small literature on this subject in other species, where individual differences in cognitive abilities have often been considered as variation around a mean (Thornton and Lukas, 2012). Yet, a similar “g factor” has been found in the other species investigated [review by Chabris (2007), see also Banerjee et al. (2009) on cotton-top tamarin *Saguinus Oedipus* and Matzel et al. (2011) on mice, but see Vonk and Povinelli (2011) and Herrmann and Call (2012) for mixed results in chimpanzees and apes, respectively]. In addition, it has been proposed that cognitive capacities correlate with brain size in rats (Anderson, 1993). In fact, although there is a growing body of research on the evolutionary significance of individual variation in cognitive abilities (Boogert et al., 2011; Cole et al., 2012; Thornton and Lukas, 2012; Cauchard et al., 2013), individual differences in cognitive abilities have still to be systematically investigated in non-human animals.

Interestingly, the mechanisms underlying species differences in cognitive abilities probably differ from those between individuals of a species. Firstly, as described above, variations of processing capacities inside a taxon cerebrotype is probably due to the cumulative effect of brain structure scaling and absolute features that go along with larger brains. Between individuals of a species, however, such kind of brain structure scaling is apparently limited (for example in the absence of hyperscaling of frontal cortex, see above). Secondly, while brain size correlates both with reaction time and general intelligence in human [see review by Chabris (2007)] macaque monkeys are faster than humans on certain visual tasks, for a degree of accuracy quite similar (Vauclair et al., 1993; Fabre-Thorpe et al., 1998; Delorme et al., 2000; Fize et al., 2011). If such a trend was confirmed with a systematic analysis (see also Washburn and Rumbaugh, 1997), this would suggest that brain size variations have different meanings given the level of variation; individuals or species. Therefore, hypotheses on the factors underlying inter and intra-species differences in cognitive abilities should be carefully examined before, maybe, being transferred between these two levels.

### Inter-individual differences in personality

The concept of “personality” describes the behavioral differences between individuals of a species that go beyond their respective differences in cognitive abilities (see Gosling, 2001, and Uher, 2011 for a review of the term used). Individual differences in “personality” can now be identified in “in a broad array of species, ranging from squid, crickets, and lizards, to trout, geese, and orangutans” (Gosling, 2008; see also Weiss et al., 2012). It is likely, however, that the neuronal mechanisms underlying individual characteristics among guppies differ from those between rats for example. Indeed, and although the differences between the two may have been overstated (Shiner and DeYoung, 2013), the traditional distinction between temperament and personality (where, schematically, temperament describes biologically anchored behavioral traits while personality includes the effects of individual construction through personal history) used in human research could be of interest here (see also Stamps and Groothuis, 2010).

Insight will come by studying the mechanisms underlying inter-individual differences in personality (Robinson, 2001; Buckholtz et al., 2007; Blatchley and Hopkins, 2010; Adelstein et al., 2011) and their genetic bases (Plomin, 1990; Fidler et al., 2007; Adams, 2011). The fact that personality traits can be linked with differences in brain anatomy (e.g., DeYoung et al., 2010) poses the question of whether personality affects brain composition, or whether brain composition affects personality. It is likely, in fact, that these two levels interact. Domestication could be particularly interesting here (Trut, 1999; Agnvall et al., 2012; Kukekova et al., 2012). As more species are studied, inter-species differences in personality structures (that is, the dimensions particular to each species onto which individual differences take place, Uher and Asendorpf, 2008) will be mapped. This is important since personality traits affect individual life-history traits (Biro and Stamps, 2008), therefore having an important role in the fitness of an individual (Dingemanse et al., 2004; Smith and Blumstein, 2008; Réale et al., 2007, 2010; Wolf et al., 2007; Schuett et al., 2010; Wolf and Weissing, 2010). In the same way that cognitive abilities cannot be studied between species without the mentality concept described earlier, inter individual differences in cognitive abilities and personality must be studied conjointly for understanding individual differences in behavior (Locurto, 2007; Carere and Locurto, 2011; Sih and Del Giudice, 2012). Both levels have been central in species evolution.

## Concluding remarks

Beside all the methodological and conceptual problems reported here, a significant bias in evolutionary neuroscience is the particular place given to human brain and cognition. As stated by Deacon; “we are, after all, the ‘sapient’ ape, distinguished from all other species by our unusual mental powers. But this has also motivated the many preconceptions that we bring to the topic that affect both the selection of scientific evidence and our interpretations of it. The single most pervasive issue behind most of these preconceptions is the notion of human intellectual superiority” (Deacon, 1990a, original quotation marks). Under this view, it is the fact that the human brain is not at the top of a criterion that makes this criterion inadequate for determining intelligence, and conversely. The misconceptions that this approach has lead, even at the brain size level (see above), have a heuristic value and warn against considering this approach for more complex variables. This comment echoes Chittka et al. (2012) who, referring to an analysis that found human species to be the slowest in a color learning task, warned that although “there may be good reasons not to equate learning speed with intelligence […] the fact that humans do not top the chart should not be one of them.”

The importance of such fallacies can be broadened to the mammalian brain in general. For instance, spontaneous mirror self-recognition occurs with the 350 g chimpanzee's brain (Gallup, 1970), the 2000 g dolphin brain *Tursiops truncatus* (Reiss and Marino, 2001) and the 4000 g elephant brain *Elephas maximus* (Plotnik et al., 2006) but also with the small 5 g magpie brain *Pica pica* (Prior et al., 2008). More generally, the complex cognitive abilities of several bird species (Emery and Clayton, 2004; Emery, 2006; Kirsch et al., 2008), suggest that the brain architecture of birds is particularly efficient. This is interesting, given the relatively recent misconception that bird intelligence was limited and their behaviors only stereotyped (Emery, 2006) and the still widely accepted postulate that the mammalian brain is the most complex and efficient structure in term of cognitive abilities. In fact, the highest ratio of cognitive abilities to neuron number could possibly be found in non-vertebrate taxa such as cephalopods (e.g., Hochner et al., 2006; Grasso and Basil, 2009; Ikeda, 2009) and insects (e.g., Menzel and Giurfa, 2001; Chittka and Skorupski, 2011).

Finally, it is particularly striking to note [as Griffin (1976) did more than twenty-five years ago] that the subjective part of behavior, that is, the way animals experience the world, has been systematically put aside in comparative studies of animal behavior. As stated by Shettleworth (2001): “it is possible, indeed usual, to study the ways in which animals acquire information about the world through their senses, process, retain and respond to it without making any commitment about the nature of their subjective experience or awareness.” Yet, what makes a bird or mammal flee danger is fear or pain, to search for food is hunger, what makes it look for mates is sexual arousal and for a place to sleep is fatigue, so that the subjective dimension of animal mind; consciousness, is the fundamental link between brain, cognition, and behavior. Studying animal brain and behavior without raising the question of how animals experience the world is likely to be as incomplete as was studying biology without evolution. In fact, this is one of evolutionary neuroscience's principal challenges.

### Conflict of interest statement

The author declares that the research was conducted in the absence of any commercial or financial relationships that could be construed as a potential conflict of interest.
